# To Clarify The Duration and Characteristics of the Continuation of Home Care for Older People With Dementia

**DOI:** 10.1093/geroni/igab046.2817

**Published:** 2021-12-17

**Authors:** Reiko Kanaya, Asuka Oyama, Hiroshi Toki, Ryohei Yamamoto, Miyae Yamakawa

**Affiliations:** 1 Osaka University Graduate School of Medicine, Suita City, Osaka, Japan; 2 Health and Counseling Center,Osaka University, Suita City, Osaka, Japan; 3 Osaka University Health and Counseling Center, Suita City, Osaka, Japan; 4 Osaka University, Suita City, Osaka, Japan

## Abstract

As populations age worldwide, older people with dementia are increasing. Caregivers are also aging, necessitating arrangements like social services. How to prolong the home care desired by older people remains unclear. Using data from the Osaka National Health Insurance Database from 2012 to 2017 on insured persons’ registers, medical notes, and care benefits, this study included 9591 people aged ≤74 years with first dementia drug prescription between April 2013 and December 2017. Using the prescription as baseline and hospitalization or nursing home admission as outcomes, home care duration and characteristics of medical and nursing care services during the year before baseline were evaluated. Survival was compared by Kaplan–Meier curves and the log-rank test. Multivariate analysis was performed using the Cox proportional hazards model. During follow-up, the outcomes were observed in 1473 patients : 317 admission , 1145 hospitalized and 11 both. Mean duration of home care in patients with the outcomes was 11.5 months, which differed significantly from patients without these outcomes. When patients were grouped by hospitalization in year before first prescription, the survival curves differed significantly. In multivariate analysis, sex, renin-angiotensin system agonists, hyperlipidemia drugs, hospitalization history in past year, care level, and diabetes drugs were significantly associated with the outcomes. Taken together, hospitalization history, female sex, and diabetes were associated with home care disruption. Those undergoing cardiovascular disease treatment continued to live at home. For people with dementia, it is important to intervene by focusing on past medical and nursing care to continue life at home.

